# Late effects in survivors of infant acute lymphoblastic leukaemia—a study of the Australian and New Zealand Children’s Haematology/Oncology Group

**DOI:** 10.1038/s41408-023-00924-5

**Published:** 2023-09-26

**Authors:** Denitza Mironova, Chitra M. Saraswati, Peter Downie, Chow Yee Lai, Eleanor Cook, Vickyanne Carruthers, Perla Moukhaiber, Fiona Molloy, Joshua Serov, Elizabeth McKinnon, Frank Alvaro, Michael Osborn, Tamas Revesz, Tim Prestidge, Siobhan Cross, Caroline M. Bateman, Andrew S. Moore, Seong Lin Khaw, Marion K. Mateos, Rishi S. Kotecha

**Affiliations:** 1 Department of Clinical Haematology, Oncology, Blood and Marrow Transplantation, Perth Children’s Hospital, Perth, Australia; 2 Leukaemia Translational Research Laboratory, Telethon Kids Cancer Centre, Telethon Kids Institute, University of Western Australia, Perth, Australia; 3 Children’s Cancer Centre, Monash Children’s Hospital, Monash Health, Clayton, Australia; 4 Children’s Cancer Centre, The Royal Children’s Hospital, Melbourne, Australia; 5 Kids Cancer Centre, Sydney Children’s Hospital, Randwick, Australia; 6 Department of Clinical Haematology and Oncology, Women’s and Children’s Hospital, North Adelaide, Australia; 7 Cancer Centre for Children, The Children’s Hospital at Westmead, Sydney, Australia; 8 Oncology Service, Children’s Health Queensland Hospital and Health Service, Brisbane, Australia; 9 Children’s Cancer and Blood Disorders, John Hunter Children’s Hospital, Newcastle, Australia; 10 Blood and Cancer Centre, Starship Children’s Hospital, Auckland, New Zealand; 11 Children’s Haematology Oncology Centre, Christchurch Hospital, Christchurch, New Zealand; 12 Discipline of Paediatrics and Child Health, School of Clinical Medicine, UNSW Medicine & Health, UNSW Sydney, Kensington, Australia; 13 Curtin Medical School, Curtin University, Perth, Australia

**Keywords:** Acute lymphocytic leukaemia, Paediatrics

**TO THE EDITOR:**

Acute lymphoblastic leukaemia (ALL) in infancy, defined as ≤1 year of age at diagnosis, is rare with an incidence of 1.8 cases per 100,000 [1]. *KMT2A*-gene rearrangements are present in up to 80% of infants with ALL and are associated with a poor prognosis. Historically, collaborative group treatment protocols have focused on intensification of conventional chemotherapy, which can include allogeneic haematopoietic stem cell transplant (HSCT) for high-risk infants, however outcomes for *KMT2A*-rearranged infant ALL have remained poor due to high relapse rates and treatment-related mortality [2, 3]. Given the rarity of the patient population and low survival rates, there is limited data regarding long-term outcomes in survivors of infant ALL in the context of receiving intensive therapy to achieve a cure.

We conducted a multi-centre retrospective analysis of all infants diagnosed with ALL in Australia and New Zealand from 1 January 1992 to 31 December 2011 (Human Research Ethics Approval RGS0000001383). Patients were identified from the cancer registry of every paediatric cancer centre in Australia and New Zealand and standardised data were captured for each patient until 28 February 2021, including demographics, disease characteristics, treatment, outcome, a systematic capture of late effects based on the Children’s Oncology Group long-term follow-up guidelines (version 5.0) and date of last follow-up. Event-free survival (EFS) was defined as the time from ALL diagnosis to treatment failure or relapse, all-cause mortality, or second malignancy. Overall survival (OS) was defined as the time from diagnosis to all-cause mortality. Time was censored at the last follow-up if no events were recorded. All analyses were performed using R (version 4.2.2).

**Fig. 1:**
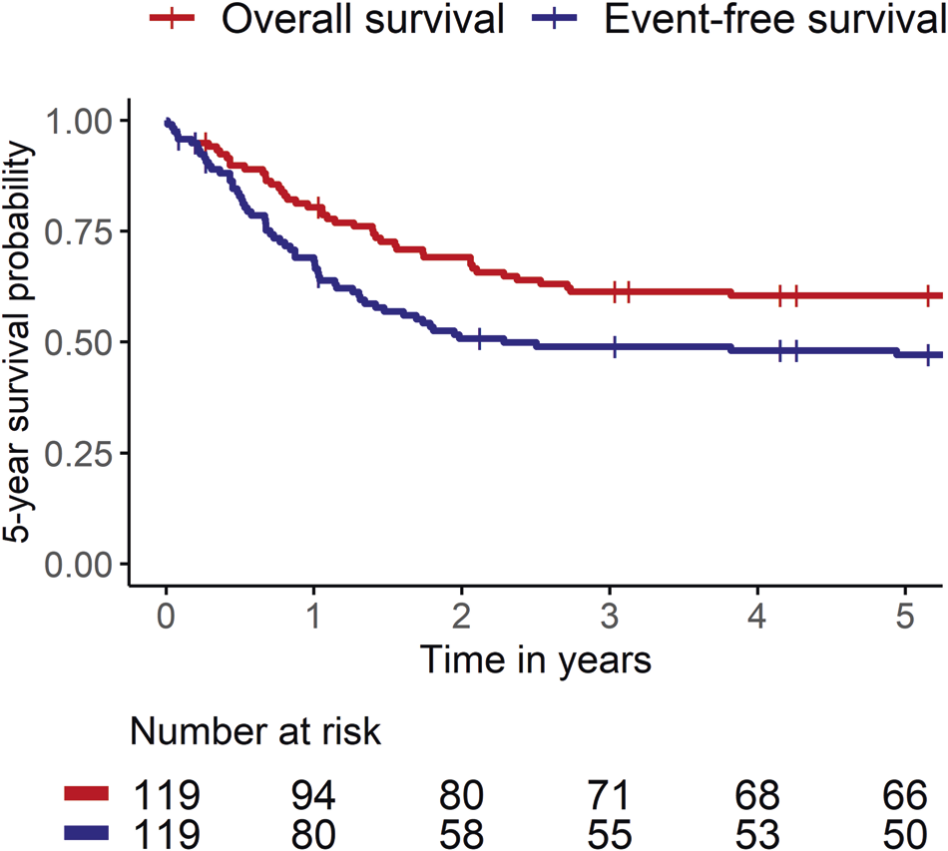
Kaplan–Meier survival curves showing 5-year event-free and overall survival of infants with acute lymphoblastic leukaemia treated in Australia and New Zealand between 1 January 1992 to 31 December 2011.

Over the 20-year period, 119 infants were diagnosed with ALL in Australia and New Zealand. Treatment was administered according to US or European-based infant-specific collaborative group protocols available at the time of diagnosis. Five-year OS was 60.5% (SE 4.5) and 5-year EFS was 47.1% (SE 4.7) (Fig. 1; Supplementary Fig. 1). EFS and OS were adversely influenced by the presence of a *KMT2A*-rearrangement, age <6 months, central nervous system (CNS) involvement, high presenting white blood cell count and CD10-negative immunophenotype, with male sex also an adverse prognostic factor for EFS on univariate analysis (Table 1; Supplementary Fig. 2). Cox regression analysis, with EFS as an endpoint, identified presence of a *KMT2A*-rearrangement, age <6 months and male sex as independent prognostic factors (Table 1). These findings highlight equivalent outcomes for infants diagnosed with ALL in Australia and New Zealand to those reported by West European/North American groups and confirm variables known to influence prognosis [2, 4, 5].

**Table 1:** Patient characteristics and analysis of prognostic factors.

Diagnostic variable	Univariate analysis				Cox-regression model	
	Patients (*n* = 119)	Events at 5 years (*n* = 61)	5-year EFS (95% CI)	*p*-value (Log-rank)	Estimated hazard ratio (95% CI)	*p*-value (Wald test)
*KMT2A* status						
Germline	38 (31.9%)	10 (16.4%)	73% (61–89)	<0.01	1	
Rearranged	69 (58.0%)	45 (73.8%)	33% (23–46)		2.29 (1.08–4.88)	0.03
Unknown	12 (10.1%)	6 (9.8%)				
*Sex*						
Female	68 (57.1%)	28 (45.9%)	58% (48–71)	<0.01	1	
Male	51 (42.9%)	33 (54.1%)	32% (21–48)		2.40 (1.35–4.26)	<0.01
*Age*						
≥6 months	70 (58.8%)	26 (42.6%)	62% (51–75)	<0.01	1	
<6 months	49 (41.2%)	35 (57.4%)	26% (16–42)		2.17 (1.12–4.19)	0.02
*CNS status*						
Negative	73 (61.3%)	30 (49.2%)	57% (47–70)	<0.01	1	
Positive	40 (33.6%)	27 (44.3%)	31% (19–49)		1.13 (0.61–2.08)	0.71
Unknown	6 (5.0%)	4 (6.6%)				
White blood cell count						
<100	53 (44.5%)	18 (29.5%)	65% (53–79)	<0.01	1	
≥100	60 (50.4%)	39 (63.9%)	34% (24–48)		1.88 (0.98–3.60)	0.06
Unknown	6 (5.0%)	4 (6.6%)				
*CD10 status*						
Positive	40 (33.6%)	14 (23.0%)	64% (50–81)	0.01	1	
Negative	76 (63.9%)	45 (73.8%)	39% (30–52)		1.04 (0.51–2.13)	0.92
Unknown	3 (2.5%)	2 (3.3%)				

Late effects data was captured for 67 patients who survived ≥5 years from diagnosis, 53 of whom survived in first complete remission. The median length of follow-up was 11.8 years (range 5.2–27.7). At least one long-term complication was observed in the majority (89.6%, *n* = 60/67), with multiple sequelae occurring in 65.7% (*n* = 44/67; two = 17; three = 13; four = 6; five = 6; six = 1; nine = 1). Within this cohort of 67 patients, late effects occurred in 86.2% (*n* = 25/29) with *KMT2A*-germline ALL and 95.2% (*n* = 20/21) of those who underwent HSCT. The one patient who underwent HSCT and did not report any sequelae had a short follow-up time of 5.4 years. On average, survivors with *KMT2A*-rearrangements suffered a greater number of late effects than those who were *KMT2A*-germline (mean 2.55 vs. 2.17) and survivors who were diagnosed <6 months of age experienced more late effects than those diagnosed >6 months of age (mean 2.78 vs. 2.20).

Neurodevelopmental complications occurred in 38.8% (*n* = 26/67). Formal neuropsychological assessment was only reported in 28.4% (*n* = 19/67), with deficits in one or more domains identified in 89.5% (*n* = 17/19). Specific developmental delays were identified in a further nine patients following assessment by allied health professionals. A variety of long-term psychological issues occurred in 32.8% (*n* = 22/67), including anxiety (*n* = 10), attention deficit hyperactivity disorder (*n* = 8), severe anger/aggression (*n* = 5), depression/self-harm (*n* = 3), substance abuse (*n* = 2) and an eating disorder (*n* = 1). Long-term seizure disorders were reported in 6.0% (*n* = 4/67).

At final follow-up, 43.3% (*n* = 29/67) had issues with weight, of whom 24 had high body mass index (BMI) (12 overweight (≥85–< 95^th^ percentile), 10 obese (≥95^th^ percentile), 2 severely obese (≥120% of 95^th^ percentile or ≥35 kg/m^2^)) and five were underweight (<5^th^ percentile). At final follow-up, 7.5% (*n* = 5/67) had short stature (<3^rd^ percentile or >2 SD below mean), one of whom received growth hormone therapy. A further five patients were treated with growth hormone leading to a final height within normal range. Pubertal delay occurred in 11.9% (*n* = 8/67) and precocious puberty in 1.5% (*n* = 1/67). Type 2 diabetes requiring medical therapy developed in 4.5% (*n* = 3/67) and hypothyroidism requiring thyroxine in 4.5% (*n* = 3/67).

Four patients (6.0%) experienced anthracycline-induced cardiomyopathy following a cumulative doxorubicin equivalent exposure ranging between 150–225 mg/m^2^. Cardiomyopathy occurred in first complete remission in two patients, both of whom subsequently received orthotopic heart transplants, and in second complete remission post-HSCT in the other two, one of whom received 12 Gy total body irradiation and a cranial boost during conditioning. This patient also developed the one second malignant neoplasm that occurred in our cohort (1.5%), namely a thyroid papillary carcinoma diagnosed 5 years post-HSCT and successfully treated with a hemithyroidectomy. Long-term respiratory complications developed in 9.0% (*n* = 6/67), comprising bronchiectasis in four patients and restrictive lung disease in two. Only one patient with long-term respiratory complications received HSCT, developing interstitial pneumonitis secondary to busulfan.

Ophthalmological complications occurred in 9.0% (*n* = 6/67), comprising bilateral cataracts post-HSCT (*n* = 2), strabismus (*n* = 2), retinal detachment (*n* = 1) and bilateral keratoconus (*n* = 1). Dentition was affected in a large proportion (38.8%, *n* = 26/67), which included combinations of hypodontia, microdontia, enamel hypoplasia, abnormal root development, over-retention of primary teeth, ectopic teeth, malocclusion and dental caries requiring extractions and restorative surgery. Ear, nose and throat issues developed in 7.5% (*n* = 5/67), with hearing loss in three patients, one of whom experienced profound bilateral hearing loss, and vocal cord dysfunction in two. A wide range of long-term gastrointestinal complications was reported in 20.9% (*n* = 14/67), including dyslipidaemia (*n* = 4), gastro-oesophageal reflux (*n* = 3), chronic diarrhoea (*n* = 2), recurrent abdominal pain (*n* = 2), oesophageal strictures requiring regular dilatations (*n* = 1), chronic graft versus host disease (GvHD) of the gut (*n* = 1), portal hypertension and oesophageal varices secondary to hepatic sinusoidal obstruction syndrome (*n* = 1), feeding issues (*n* = 1) and non-alcoholic steatohepatitis (*n* = 1). Urinary tract dysfunction occurred in 14.9% (*n* = 10/67), with nocturnal enuresis the predominant finding (*n* = 5). Three patients (4.5%) suffered dermatological toxicity, including alopecia (*n* = 2) and severe scleroderma secondary to chronic GvHD (*n* = 1). One patient experienced a large unprovoked iliofemoral venous thromboembolism 14.3 years following the completion of therapy.

The rarity of the patient population coupled with historically poor outcomes has led to a paucity of data regarding late effects in survivors of infant ALL [6–12]. The high incidence in our cohort is in keeping with previous reports which identified late complications in approximately three-quarters of all survivors [6, 7]. Growth failure, BMI abnormalities, gonadal dysfunction and hypothyroidism comprised the predominant sequelae in prior studies [6–9]. Additionally, a high burden of neurodevelopmental issues was identified in three small studies in which the majority of patients received radiation [9–11]. Whilst we also observed a high incidence of increased BMI, growth failure was less frequent and may be reflected by the low number of survivors who received radiation in our study (*n* = 7/67; total body irradiation = 4, cranial = 3, testicular = 2). However, despite the low incidence of cranial radiation in our cohort, there was a significant burden of neurodevelopmental issues, which is in contrast to a study that identified positive neurodevelopmental outcomes following formal assessment of infants treated with high-dose methotrexate as CNS-directed therapy rather than radiation [12]. However, most of the children in this study were of pre-elementary school age at the time of testing, and thus long-term risk was not captured, indicating the need for formal assessment beyond 5 years of follow-up. Indeed, the incidence of neurodevelopmental issues in our cohort is likely to be underestimated given the low number that underwent formal assessments. Our study is also likely to underestimate cardiac dysfunction, as the study design identified patients with severe and clinically significant anthracycline-induced cardiomyopathy and not those with subclinical dysfunction on serial echocardiograms or masked dysfunction which would only become evident in the context of physiological stress such as pregnancy. Nonetheless, the high incidence of serious cardiac complications despite relatively low cumulative anthracycline exposures, warrants further discussion regarding standardising the use of dexrazoxane for infants who are exposed to anthracyclines [13]. Finally, we also report a high incidence of late effects in infants with *KMT2A*-germline ALL. This is in contrast to a previous study that did not report significant long-term complications in their *KMT2A*-germline population, however, this observation was limited by a short duration of follow-up [8]. Our finding is important as infants with *KMT2A*-germline ALL have relatively good outcomes with current intensified therapy and thus represent ideal candidates in whom to consider de-escalation of therapy within a clinical trial setting to reduce the risk of long-term sequelae [3].

While this study is limited by its retrospective nature and inherent heterogeneity of treatment protocols used over time, it provides evidence that the intensive treatment offered to infants with ALL leads to long-term complications in the majority of survivors. A structured and targeted prospective approach for surveillance and treatment of late effects in a systematic and longitudinal manner, including evaluation of neurocognitive and adaptive outcomes, should be implemented as a component of future collaborative group therapeutic protocols. The advent of novel immunotherapeutic approaches in combination with chemotherapy holds significant promise for improving the survival outcomes of infants with ALL [14], thus providing greater imperative to recognise potential late effects and allow for early intervention to reduce the risk of long-term sequelae in this vulnerable cohort.

### Supplementary information

Supplementary Figures
