# Supplementary material for: Late effects in survivors of infant acute lymphoblastic leukaemia—a study of the Australian and New Zealand Children’s Haematology/Oncology Group
Source: Blood Cancer J. 2023 Sep 26;13(1):150. doi: 10.1038/s41408-023-00924-5 (PMC10522653; doi:10.1038/s41408-023-00924-5)
Supplement: Supplementary file 1 — Supplementary Figures [file 41408_2023_924_MOESM1_ESM.pdf]

Supplementary Figure 1. Kaplan-Meier survival curve showing 20-year event-free and overall survival of infants with acute lymphoblastic leukaemia treated in Australia and New Zealand between 1st January 1992 to 31st December 2011.

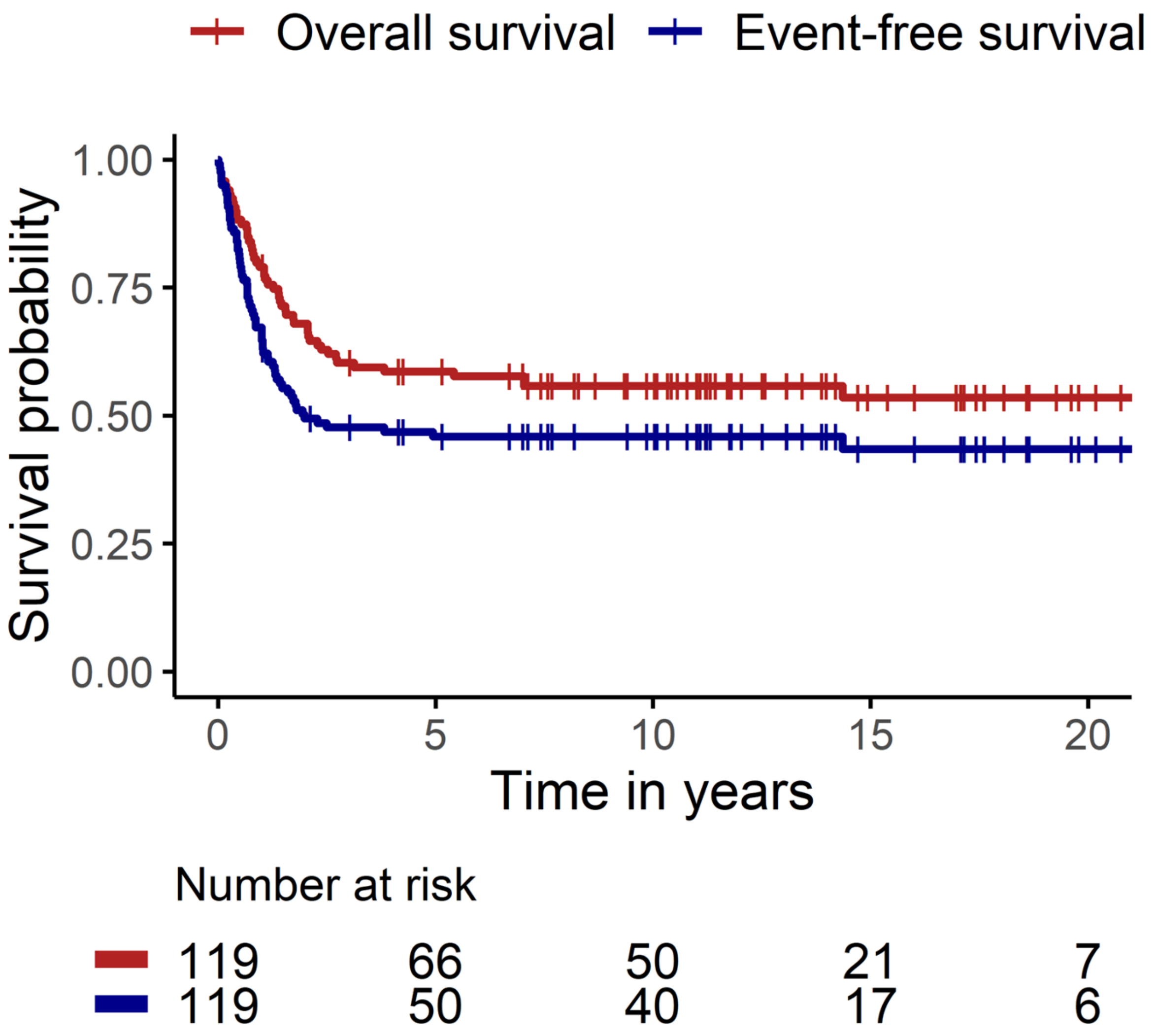

Supplementary Figure 2. Kaplan-Meier survival curves showing event-free (left panel) and overall survival (right panel) of infants with acute lymphoblastic leukaemia treated in Australia and New Zealand between 1st January 1992 to 31st December 2011 stratified by (A) *KMT2A* status (B) Sex (C) Age (D) CNS status (E) White Blood Cell count and (F) CD10 status at diagnosis.

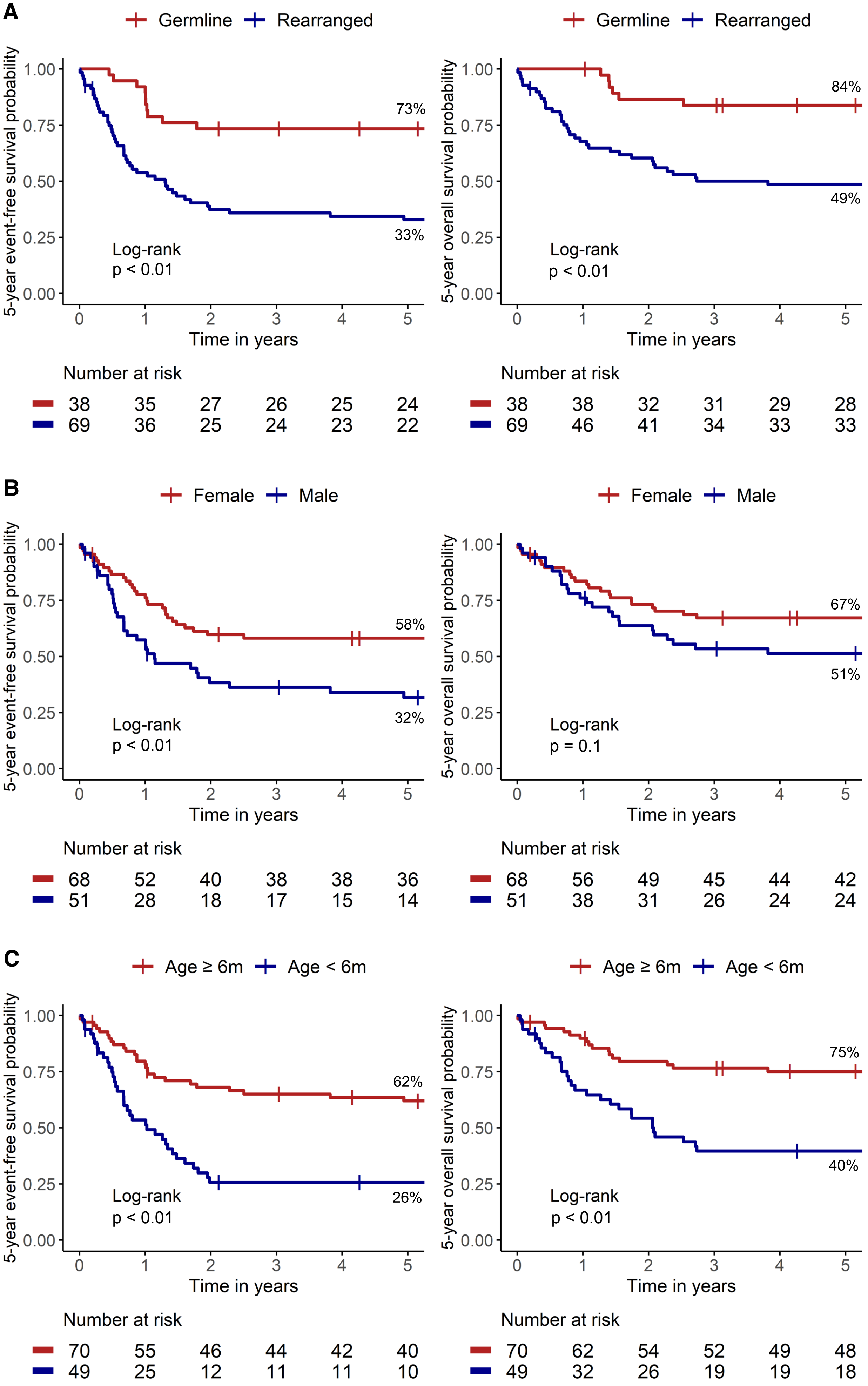

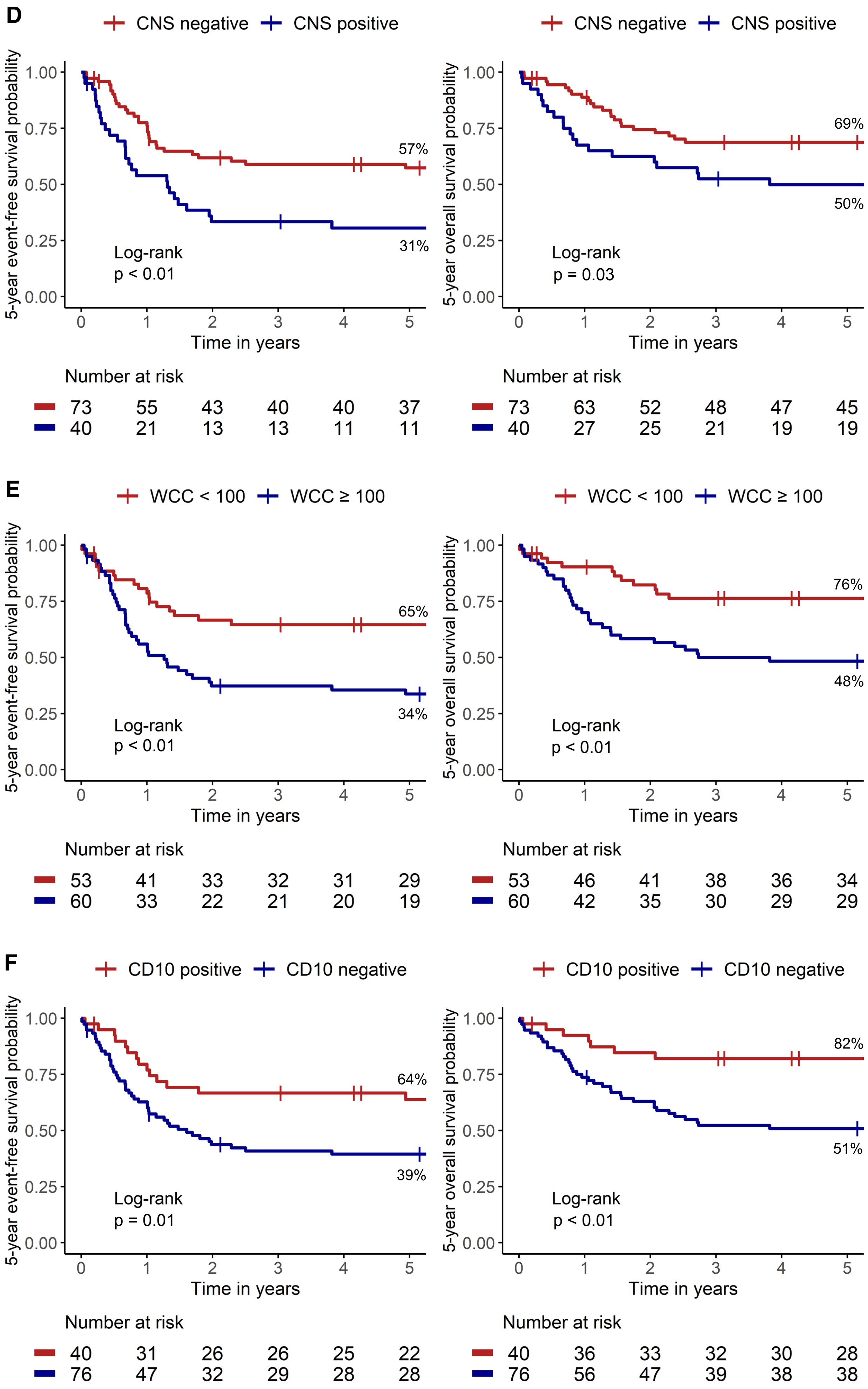

D
CNS negative
CNS positive
5-year event-free survival probability
5-year overall survival probability
1.00
0.75
0.50
0.25
0.00
0 1 2 3 4 5
Time in years
57%
31%
Log-rank
p < 0.01
69%
50%
Log-rank
p = 0.03
Number at risk
73 55 43 40 40 37
40 21 13 13 11 11
73 63 52 48 47 45
40 27 25 21 19 19
E
WCC < 100
WCC ≥ 100
65%
34%
Log-rank
p < 0.01
76%
48%
Log-rank
p < 0.01
Number at risk
53 41 33 32 31 29
60 33 22 21 20 19
53 46 41 38 36 34
60 42 35 30 29 29
F
CD10 positive
CD10 negative
64%
39%
Log-rank
p = 0.01
82%
51%
Log-rank
p < 0.01
Number at risk
40 31 26 26 25 22
76 47 32 29 28 28
40 36 33 32 30 28
76 56 47 39 38 38
